# Is periodontitis a risk indicator for cancer? A meta-analysis

**DOI:** 10.1371/journal.pone.0195683

**Published:** 2018-04-17

**Authors:** Stefano Corbella, Paolo Veronesi, Viviana Galimberti, Roberto Weinstein, Massimo Del Fabbro, Luca Francetti

**Affiliations:** 1 IRCCS Istituto Ortopedico Galeazzi, Milan, Italy; 2 Department of Biomedical, Surgical and Dental Sciences, Università degli Studi di Milano, Milan, Italy; 3 European Institute of Oncology, Division of Senology, Milan, Italy; 4 Università degli Studi di Milano, Milan, Italy; 5 Scientific Director D&S ICH Humanitas Dental Center, Rozzano, Milan, Italy; Boston University Henry M Goldman School of Dental Medicine, UNITED STATES

## Abstract

**Background:**

The aim of the present systematic review was to evaluate the hypothesis of an association between periodontitis and the development of cancer.

**Methods:**

Two reviewers independently screened electronic and manual sources for pertinent articles. Primary outcome measures were the occurrence of neoplasm diagnosis in exposed and non-exposed groups, reported to evaluate association between cancer and periodontitis.

**Results:**

Of the 490 initially retrieved papers 10 were included in the qualitative synthesis and eight in the quantitative synthesis; the eight papers covered six studies. Considering hazard ratios, a statistically significant association was found for all cancers studied (1.14; CI 95%: 1.04, 1.24), digestive tract cancer (1.34; CI 95%: 1.05, 1.72), pancreatic cancer (1.74; CI 95%: 1.21, 2.52), prostate cancer (1.25; CI 95%: 1.04, 1.51), breast cancer (1.11; CI 95%: 1.00, 1.23), corpus uteri cancer (2.20; CI 95%: 1.16, 4.18), lung cancer (1.24; CI 95%: 1.06, 1.45), hematological cancer (1.30; CI 95%: 1.11, 1.53), esophagus / oropharyngeal cancer pooled together (2.25; CI 95%: 1.30, 3.90) and Non-Hodgkin lymphoma (1.30; CI 95%: 1.11, 1.52).

**Conclusions:**

Despite the sparse scientific evidence and considering the low statistical power of the results, this systematic review revealed a substantial lack of studies with standardized and comparable methods to speculate about the association between periodontitis and cancer; more studies are need in order to explore further the scientific evidence of such correlation.

## Introduction

Periodontal diseases and, in particular, periodontitis is reported to be potentially associated with some systemic diseases and conditions such as cardiovascular disease, the impairment of glycemic control in patients with diabetes and preterm births or low-birth weight [1–6]. Such correlation could be due to several mechanisms: 1) the spread of bacteria from the oral cavity could cause tissue damage to various organs [7, 8]; 2) the increase in inflammatory systemic burden [4, 9, 10], that may augment the susceptibility of atheromatous plaque formation [7]; 3) an autoimmune response which could be triggered by bacterial epitopes from oral bacterial species [7].

Following the publication of some primary reports [11, 12], the authors hypothesized that periodontitis could be an independent risk factor for cancer development (both locally and at a distance) due to the long-standing chronic inflammatory status of the periodontal tissues [13, 14]. Some mechanisms were advocated explaining the potential basis of such association. Published studies demonstrated a role of viruses such as Human Papilloma virus (HPV) and Epstein-Barr virus (EBV), that could be detected in periodontal pockets, as suspected agents for oral cancer through the activation of specific oncogenes (such as E6 and E7 for HPV) [15–17]. Specific pathogens, such as *P*. *gingivalis*, were demonstrated to prevent, after invading the epithelium, cell apoptosis, thus favoring cancer initiation [18–20]. These pathogens could be found in carcinomas of the gingiva [18], but could also be associated with distant tumors [21].

Indirect mechanisms for a link between periodontitis and cancer were mainly related to the known association between the inflammatory process itself and cancer [22–24]. Indeed, it was demonstrated that periodontitis may induce a significant increase in inflammatory markers and molecules that enhances the inflammatory reaction. This condition causes the release of reactive oxygen species and other metabolites that could promote cancer initiation [22, 24]. Moreover, the stimulation of the inflammatory process and the presence of cell-stimulating signals may create an optimal environment for cell proliferation and differentiation [22, 24]. Such mechanism could act both locally and at a distance [22, 24]. Furthermore, other authors hypothesized that a para-inflammation mechanism (a low-grade inflammation that could be associated to periodontitis [25]) can be involved in cancer development [26].

Published systematic reviews of the literature have investigated the association between periodontitis and oral cancer [23, 27]. Even though a positive correlation was found in one meta-analysis, the validity of the results was limited by the criteria adopted for periodontal assessment in the included studies [28]. Another systematic review of the literature, published by Fitzpatrick and Katz in 2010, found a positive association between periodontitis and any type of cancer, although this was only a qualitative analysis of the included papers [13]. To our knowledge, a comprehensive review of the literature with meta-analysis is missing in the literature and, for this reason, the present study was carried out.

The aim of the present systematic review of the literature was to evaluate if, in humans (P), having periodontitis (I) (compared to being periodontally healthy (C)) implies a higher risk of neoplasms (O).

## Materials and methods

The study protocol was approved by the Review Board of the Center for Research in Oral Implantology of the “Università degli Studi di Milano” in Milan, Italy in January 2016. The protocol was registered in PROSPERO (http://www.crd.york.ac.uk/PROSPERO) before the beginning of the research with the number CRD42016036061.

The study was reported following the instructions of the Preferred Reporting Items for Systematic Review and Meta-analysis (PRISMA) statement [29] and was conducted according to the Cochrane Handbook [30].

### PICO question

In human subjects, does having periodontitis (compared to being periodontally healthy) increase the risk of neoplasms initiation (P: human subjects; I (Indicator): Periodontitis; C: No periodontitis; O: Cancer)?

### Search strategy

An electronic search of the following databases was conducted: MEDLINE / PubMed, Scopus, ISI Web of Science, Cochrane Central and EMBASE using an ad hoc created search string obtained combining pertinent keywords with the use of boolean operators “OR” and “AND”. The search string for PubMed was: ("periodont*"[All Fields] OR "periodontal disease*"[All Fields]) AND ("cancer*"[All Fields] OR "oncolog*"[All Fields] OR "leukoplakia"[All Fields] OR "eritroplakia"[All Fields]). Grey literature was also searched (Greylit, OpenGrey). The reference list of the included papers and the table of contents of Journal of Clinical Periodontology, Journal of Periodontology, Journal of Periodontal Research, Journal of Dentistry, Journal of Dental Research, CA—A Cancer Journal for Clinicians, Nature Reviews Cancer, The Lancet Oncology, Journal of Clinical Oncology, Annals of Oncology, Clinical Cancer Research, and European Journal of Cancer were manually searched beginning from 2000. The last electronic search was performed September, 20th 2017.

### Selection criteria

Two authors (SC, MDF) independently screened titles and abstracts and then full texts evaluating them for potential inclusion on the basis of the following selection criteria:

1. Studies on human subjects
2. Case-control and prospective cohort studies
3. Studies in which data about cases (subjects who developed neoplasms) and controls (subjects who did not develop neoplasms) could be distinguished and extrapolated
4. Clear definition of periodontitis
5. Description of how confounders were controlled in the analysis (adjustments)

The level of concordance, calculated through Cohen’s kappa, between the two reviewers was 0.92 for titles and abstracts and 0.98 for full texts.

In case of disagreement in the article selection process a third reviewer (LF) was asked to decisively solve the discussion.

### Data extraction

Two reviewers (SC, LF) independently collected the following data from the studies included:

Author names, year of publication, country of examination, sample characteristics (ethnicity, mean age, smoking status, alcohol consumption), definition of periodontal disease (periodontitis), type of neoplasia, outcome measure (the diagnosis of a neoplasm in the exposed and in non-exposed group) and parameters for adjustment.

If the information provided in the paper was insufficient, the corresponding author of the article would have been contacted for the missing data. However, all studies provided sufficient information about outcomes.

### Quality assessment for included studies

The quality assessment of the included study was performed using the tool for quality assessment of case-control and cohort studies elaborated by the National Institute of Health—National Heart, Lung, and Blood Institute (https://www.nhlbi.nih.gov/health-pro/guidelines/in-develop/cardiovascular-risk-reduction/tools/cohort). The tool was designed to assess the research question, the characteristics of study population, the recruitment procedure and sample size justification, if the exposure was assessed prior to outcome measurement, if the timeframe was sufficient, the characteristics of the exposure, how outcomes were recorded, the follow-up rate and the statistical analysis. Such tool was independently used for assessment by the two reviewers (SC, RW) (k = 0.93).

Studies that scored “No” for one or no items were judged having good quality, those that score “No” for more than one but less than three items were judged having fair quality. Other studies were judged of poor quality and were excluded from the quantitative analysis.

### Summary measures, synthesis of the results and additional analysis

The synthesis of the results was performed with the use of the software RevMan (Review Manager Version 5.3, 2014; The Nordic Cochrane Center, The Cochrane Collaboration, Copenhagen, Denmark).

For studies presenting data on the same cohort with different follow-up, only the report with the longer time of observation was considered for the quantitative synthesis. The most adjusted model (and outcome measure) was used when more models were presented in the same study.

The measure of the association between cancer and periodontitis (hazard ratio (HR) which is the hazard in the exposed groups divided by the hazard in the non-exposed groups and relative 95% confidence interval (CI) (an interval estimator)) was extrapolated from the papers presenting it. In studies in which such parameter was not reported it would have been calculated through appropriate method. However, all included papers presented HRs.

In the meta-analysis, in order to estimate the association between periodontitis and cancer in adult subjects, the method of inverse variance was used combining the results using the DerSimonian and Laird’s random-effect model [31] and the Mantel-Haenszel fixed-effect model [32]. The analysis was performed using as the summary measure pooled HR. For each measure, pooled estimate of 95% CI was calculated. Standard error (SE) was computed as follows: SE = ((ln (Upper CI) / ln (Lower CI)) / 3.92).

The consistency of the results was measured using Cochran’s test considering it significant if P < 0.1. The quantification of such heterogeneity was computed through I^2^ statistics, that served to describe the total variation across studies that was due to heterogeneity rather than to chance. If I^2^ was found to be less than 40% the heterogeneity was negligible, if it was found from 40% to 60% it signified a moderate heterogeneity, if from 60% to 90% it signified a substantial heterogeneity while it showed a considerable heterogeneity if it was from 75% to 100% [30].

One single analysis was performed for each type of cancer as it was defined in the included studies. In case one single study was present for one type of cancer, a meta-analysis could not be performed and the results of the study were extracted and presented.

Sensitivity analysis was performed by substituting one paper with a less recent one when belonging to the same study, or substituting one outcome value in one study with another one with less adjustments.

## Results

### Study selection

**Fig 1:**
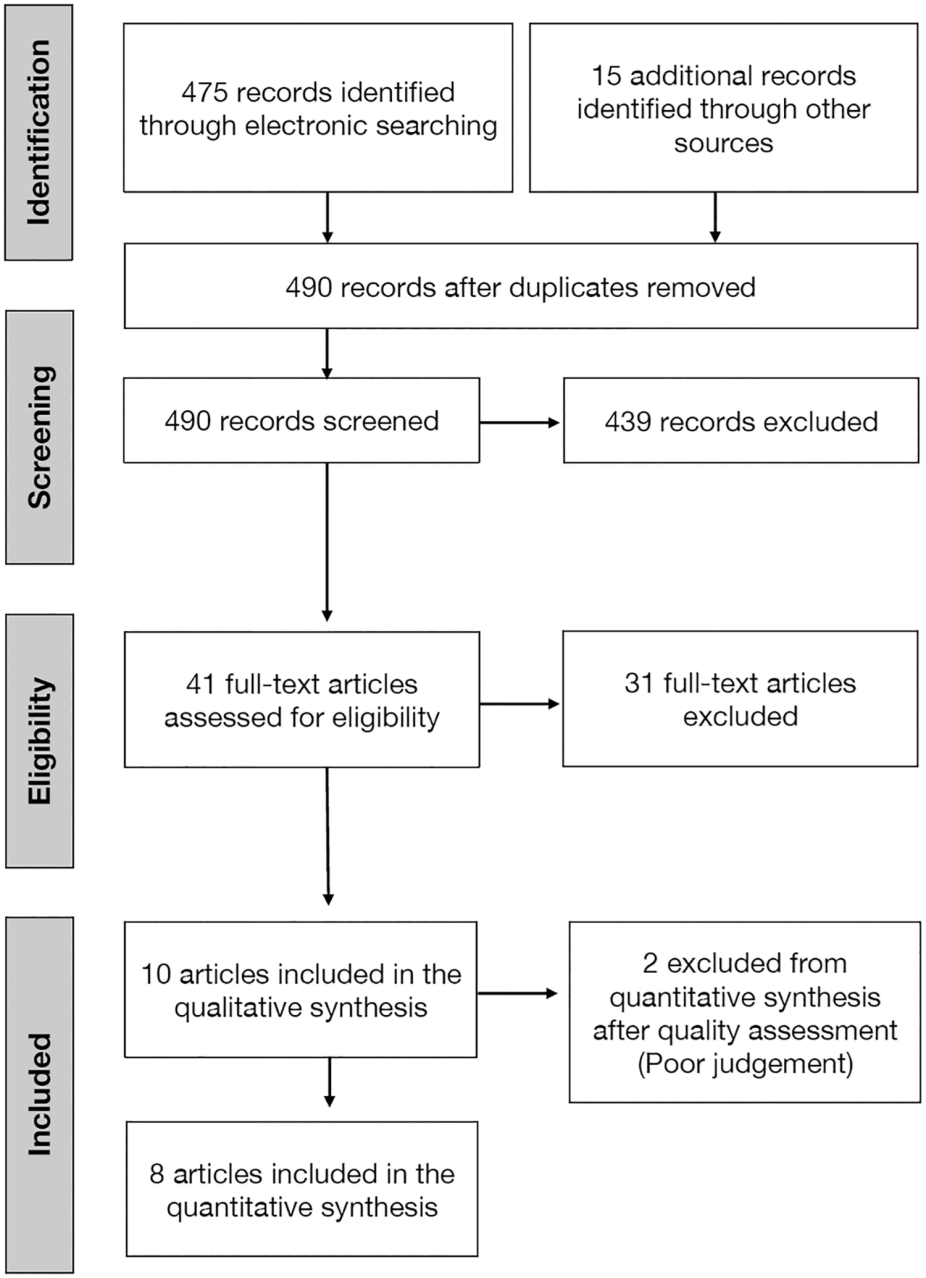
Diagram of article selection process.

The flowchart of article selection process is shown in Fig 1. The literature search resulted in the identification of 475 papers from electronic databases and 15 from a manual search. Of the 490 titles and abstracts retrieved, 439 papers were excluded as they were inconsistent with the aims of the review. A total of 41 full texts were assessed for eligibility; after screening for inclusion criteria 31 papers were excluded (the reasons for exclusion are presented in Table 1) and 10 papers (referring to 6 studies) were finally considered in the review.

**Table 1:** Excluded papers and reasons for exclusion.

Studies	Reason for exclusion
Abnet et al. 2001, Abnet et al. 2005a, Abnet et al. 2005b, Abnet et al. 2008, Ansai et al. 2013, Bundgaard et al. 1995, Divaris et al. 2010, Fernandez-Garrote et al. 2010^a^, Hiraki et al. 2008, Marshall et al. 1992, Stolzenberg et al. 2003, Talamini et al. 2000, Tu et al. 2007, Watabe et al. 1998, Wei et al. 2005, Zheng et al. 1990	No periodontitis but tooth loss
Ahn et al. 2012	Narrative review
Cabrera et al. 2005	Association between poor oral health and cardiovascular disease
Chang et al. 2016	Association between all periodontal diseases (including gingivitis) and pancreatic cancer
Demirer et al. 1990, Guha et al. 2007, Rosenquist et al. 2005, Sepehr et al. 2005, Talamini et al. 2000	Association between oral health in general and cancer
Hujoel et al. 2003	Association between periodontitis and mortality for cancer
Mai et al. 2015	Association between periodontal pathogens and cancer
Mai et al. 2016, Salazar et al. 2012, Soder et al. 2011, Tezal et al. 2005, Tezal et al. 2007	Unclear / No definition of periodontitis
Yen et al. 2014	Other association

^a^ No periodontitis but tooth loss and gingival bleeding

### Study characteristics

The main characteristics of the six included studies are presented in Table 2. One study was set up in Sweden [33], and five in the United States [34–42]. With regard to characteristics of the population, one study was composed of twins from the Swedish Twin Registry [33] and one was composed on people from nine medical facilities in the area of Boston [35]. Two studies were case-control [35, 38] and this should be considered as a potential source of methodological heterogeneity among the included papers.

**Table 2:** General characteristics of the included studies.

Study ID	Authors	Year	Country	Population characteristics	Cancer type	Periodontal assessment
#1	Arora et al.	2010	Sweden	55% female; Twins from Swedish Twin Registry (n = 15,333)	Any cancer; Digestive tract; Colorectal; Pancreas; Stomach; Bladder; Prostate; Breast; Corpus Uteri; Lung	Self-assessed in 1963 through a questionnaire: “Have you noticed that some of your own teeth have come loose or fallen out on their own?”
#2	Eliot et al.	2013	USA	Nine medical facilities in the Boston (USA) area (n = 1,080)	Oral cavity; Pharynx; Larynx	Self-assessed and self-reported
#3	Mai et al.	2014	USA	Womens’s Health Initiative Observational Study; Postmenopausal women; Mean age 48.3 years; (n = 93,676)	Lung	Self-assessed asking the question: “Has a dentist or dental hygienist ever told you that you had periodontal or gum disease?” [42]
	Freudenheim et al.	2015			Breast	
#4	Michaud et al.	2007	USA	Health Professionals Follow-up Study (57.6% dentists) (n = 51,529)	Pancreas	Self-assessed asking the question: “Have you had periodontal disease with bone loss?” [43, 44]
	Michaud et al.	2008			Any cancer; Lung; Oropharynx; Esophagus; Stomach; Pancreas; Colon-Rectus; Kidney; Bladder; Prostate; Hematopoietic; Brain; Melanoma	
	Michaud et al.	2016			Any cancer; Prostate; Colon-Rectus; Melanoma; Bladder; Lung; Kidney; Esophagus and oropharynx; Pancreas	
	Bertrand et al.	2017			Non-Hodgkin lymphoma	
#5	Momen-Heravi et al.	2017	USA	Nurses’ Health Study (n = 77,443)	Colorectal cancer	Self-reported asking the question “Have you had periodontal bone loss diagnosed by a physician?”
#6	Mazul et al.	2017	USA	Carolina Head and Neck Cancer Study (n = 492)	Head and Neck Squamous Cell Carcinoma	History of “gum disease diagnosed by a dentist”

Two of the included articles [36, 37] reported data from the Women’s Health Initiative Observational Study (WHIOS), that enlisted 93,676 postmenopausal women. Four papers [34, 39–41] reported data from the Health Professionals Follow-up Study (HPFS) that is composed of 51,529 men in health professions (57.6% dentists). One article [42] was based on data from the Nurses’ Health Study (NHS) and one [38] from the Caroline Head and Neck Cancer Study (CHNCS). HR was used as outcome measure in eight papers [33–37, 40–42], OR was used in two papers [35, 38] and RR in one paper [39]. Outcome measures and adjustments are shown in Table 3. Two studies examined population that was composed exclusively by women (WHIOS and NHS), one exclusively by men (HPFS) and two studied a population composed both by men and women (CHNCS and Swedish Twin Registry). Differences in the studied population could be considered as a further source of heterogeneity.

**Table 3:** Outcomes and adjustments.

Study ID	Authors	Year	Outcomes	Adjustments
#1	Arora et al.	2010	HR	Gender, age, education, employment, number of siblings, smoking status, smoking status of partner, alcohol status, diabetes, body mass index
#2	Eliot et al.	2013	OR	Age, Gender, Race, Smoking, Alcohol status, education, annual household income
#3	Mai et al.	2014	HR	Unadjusted; Age; MODEL A: Age, smoking status, pack-years; MODEL B: MODEL A + education, race, BMI, alcohol status, hormone use, dental visits, physical activity, region of residence, aspirin use, secondhand smoke
	Freudenheim et al.	2015		Age; MODEL 1: Age, Education, Race, BMI, Age at menarche, Age at menopause, Parity, Age at first birth, Hormone use, Alcohol status, Physical activity, NS Anti-Inflammatory Drugs; MODEL 2: MODEL 1 + Smoking status, pack-years
#4	Michaud et al.	2007	RR	Age; MODEL A: Age, smoking history, profession, race, geographic location, history of diabetes, BMI, height, history of cholecystectomy, Nonsteroideal anti-inflammatory drug use, multivitamin use, baseline teeth numbers; MODEL B: dietary intakes of fruits and vegetables, vitamin D, calcium, sucrose, and total calories
	Michaud et al.	2008	HR	MODEL A: Age, race, physical activity, diabetes, alcohol status, BMI, geographical location, height, calcium intake, red-meat intake, fruit and vegetables intake, vitamin D score; MODEL B: MODEL A + smoking history, pack-years
	Michaud et al.	2016		Age, Race, Alcohol status, physical activity, diabetes, BMI, geographical location, height, NSAID use
	Bertrand et al.	2017		Age, Race, Diabetes history, BMI at baseline, geographical location, smoking, NSAID use
#5	Momen-Heravi et al.	2017	HR	Age, race, smoking, history of colorectal cancer in a parent or sibling, history of sigmoidoscopy / colonscopy, current physical activity, regular aspirin use, multivitamin use, type 2 diabetes, alcohol consumption, adult BMI, energy-adjusted intake of total calcium, vitamin D, folate, red meat and processed meat and postmenopausal hormone use
#6	Mazul et al.	2017	OR	Age, race, sex, alcohol use, socioeconomic status (income, insurance, education)

HR: Hazard Ratio; OR: Odds Ratio; COPD: Chronic Obstructive Pulmonary Disease; BMI: Body Mass Index; NSAID: Non-Steroideal Anti-Inflammatory Drug

With regard to the methods of assessing the presence of periodontitis, in one paper periodontal status was assessed once in 1963 by a question [33], it was self-reported and self-assessed in another study [35] but none of the questionnaires used for these studies were validated before. In one study, periodontal status was explored asking if a “gum disease” was ever diagnosed by a dentist [38]. In the WHIOS the questionnaire used to evaluate the presence of periodontal disease was previously validated by LaMonte and coworkers in 2014 [43]. In the HPFS, the questionnaire used was validated both in non-professionals [44] and in dental professionals of the same cohort [45]. In one paper [42] the same questionnaire of HPFS was used but it was not specifically validated for the NHS cohort.

Cancer types were classified according to the International Classification of Diseases (ICD) Ninth Edition in most of the included papers [33, 35–37, 42]. In one study cases with cancer were selected from Swedish National Cancer Register [33]. In other studies cancer type was assessed through a questionnaire that was then confirmed by medical records [34, 36, 37, 39–42].

### Quality assessment

A summary of the results of the quality assessment of the included studies is presented in Table 4. Two papers were judged, on the basis of the considered parameters, to be of poor quality and were excluded from the quantitative synthesis [35, 38]; both of them did not assess if the exposure (periodontitis) was present before the development of cancer.

**Table 4:** Summary of quality assessment.

Study ID	Authors	Year	Quality rating	Reason for downgrading
#1	Arora et al.	2010	Fair	Definition / assessment of periodontitis not validated Periodontal conditions measured once
#2	Eliot et al.	2013	Poor	No sample size justification Definition / assessment of periodontitis not validated Periodontal conditions measured once
#3	Mai et al.	2014	Good	-
	Freudenheim et al.	2015	Good	-
#4	Michaud et al.	2007	Good	-
	Michaud et al.	2008	Good	-
	Michaud et al.	2016	Good	-
	Bertrand et al.	2017	Good	-
#5	Momen-Heravi et al.	2017	Fair	No sample size justification Definition / assessment of periodontitis not validated
#6	Mazul et al.	2017	Poor	No sample size justification Definition / assessment of periodontitis not validated Periodontal conditions measured once

### Results of the quantitative analysis

The results of the meta-analysis are summarized in Table 5. A statistically significant association was found considering all cancers, digestive tract cancer (evaluated in the study by Arora and coworkers; HR = 1.34 [1.05, 1.72] [33]), pancreatic cancer, prostate cancer, breast cancer, corpus uteri cancer (evaluated in the study by Arora and coworkers; HR = 2.20 [1.16, 4.18] [33]), lung cancer, hematological cancer (evaluated in the study by Michaud and coworkers published in 2008; HR = 1.30 [1.11, 1.53] [41]), and esophagus / oropharyngeal cancer (evaluated in the study by Michaud and coworkers in 2016; HR = 2.25 [1.30, 3.90] [40]) pooled together and Non-Hodgkin lymphoma (evaluated by Bertrand and coworkers; HR = 1.30 [1.11, 1.52] [34]). The heterogeneity was negligible or its evaluation was not applicable if only one study was included in the meta-analysis.

**Table 5:** Summary of the results.

Cancer		Outcome	N° of Studies	Value [95% CI]	Test for overall effect P	I^2^
*Any cancer*						
		HR	2	1.14 [1.04, 1.24]	0.004	0%
*Colon—rectus*						
	Fair quality	HR	2	0.90 [0.73, 1.11]	0.42	0%
	Good quality	HR	1	1.03 [0.76, 1.40]	0.85	N/A
	All	HR	3	0.94 [0.79, 1.12]	0.49	0%
*Pancreas*						
		HR	2	1.74 [1.21, 2.52]	0.003	0%
*Stomach*						
		HR	2	1.03 [0.71, 1.48]	0.90	0%
*Bladder*						
		HR	2	1.31 [0.93, 1.84]	0.12	0%
*Prostate*						
		HR	2	1.25 [1.04, 1.51]	0.02	16%
*Breast*						
		HR	2	1.11 [1.00, 1.23]	0.04	0%
*Lung*						
	*Fair quality*	*HR*	*1*	1.41 [0.81, 2.46]	0.23	N/A
	*Good quality*	*HR*	*2*	1.22 [1.04, 1.44]	0.58	0%
	All	HR	3	1.24 [1.06, 1.45]	0.007	0%

CI: Confidence Interval; HR: Hazard Ratio; N/A: Not applicable

### Sensitivity analysis

Sensitivity analysis did not find any changes in the evaluation of the association between periodontitis and cancers. One exception was found in the association between periodontitis and esophageal cancer, as it was evaluated in the paper by Michaud and co-workers published in 2008 [41]. Considering the HR obtained after some adjustments (model A, see Table 3) a significant association was found while it was not significant using other adjustments (model B).

## Discussion

The present systematic review of the literature found a small, but statistically significant association, between the diagnosis of periodontitis and the presence of cancer. The meta-analysis performed considering HRs as outcomes found an association between periodontitis and the presence of any type of cancer as well as the presence of specific neoplasms such as digestive tract cancer, pancreatic cancer, prostate cancer, breast cancer, corpus uteri cancer, lung cancer, hematological cancer, and esophagus / oropharyngeal cancer pooled together and Non-Hodgkin lymphoma.

In order to interpret adequately the validity of the obtained results, several limitations of the study should be considered.

One important limitation is the criteria of selection of the included papers. In order to remain adherent to the aim of the review we included only papers evaluating patients with periodontitis. Studies that correlate cancer with tooth loss, to the amount of attachment loss or to other clinical measures were excluded because such parameters could be modified also by clinical conditions other than periodontitis. As an example, tooth loss could be caused by a number of factors such as caries, root fractures, infection of endodontic origin, and dental or maxillary trauma [46–48]. Tooth loss could also be associated to low socioeconomic status that is also considered as an important risk factor for the development of cancer in general and oral cancer particularly [49–52]. Then, considering the definitions and classification schemes used for periodontitis in included studies, several considerations should be made. The use of self-reported periodontitis, even though validated [43–45], could be considered a significant bias because they are based substantially on subjective perception. However, it should be considered that one of the used questionnaire had a 0.78 and 0.76 positive predictive values (respectively among dentists and non-dentist health professionals) [44, 45] and another one showed a moderate accuracy to characterize periodontal disease prevalence [43]. In general, most of the included studies provided an insufficient description of the methods used for classification, when performed by a dental specialist. In one study the authors classified cases of periodontal diseases using the distance between the cemento-enamel junction and the bone crest, as evaluated through periapical radiographs [53]. The absence of a validation of this method and of the description of which threshold was use to define cases causes the exclusion of the paper from the present review. Another study by Tezal and colleagues related oral cancer to the presence of sites with clinical attachment loss higher than 1.5 mm [12]. One cross-sectional study found a positive correlation between the level of bleeding on probing and gastric precancerous lesions [54].

Another issue to be considered is the outcome measure. Hazard ratios were used in most of the considered epidemiological studies even though a criticism was raised about the value of HR as an indicator of a causal relationship between two conditions [51]. Other studies used ORs as outcome measures, aiming at measuring the association between exposure and outcome. Differently from HRs, which represent a point estimate, ORs derive from *post hoc* calculation. Considering this, the two outcomes could not be pooled in the meta-analysis, thus reducing the number of papers available for each comparison. However, none of the studies included in the quantitative synthesis presented the results as ORs.

The relatively strict inclusion criteria used in the present study have considerably limited the number of studies available for the meta-analysis and this was because we chose to select only studies that were comparable. Indeed, for two cancer types (colon-rectus, and lung) meta-analysis included three papers, for six types (all cancers, stomach, bladder, prostate, pancreas and breast) the meta-analysis included two papers, and for other types just one paper for each was available. Although some authors it was stated that two papers could be sufficient to perform a meta-analysis [55], we should consider the number of available studies as a limitation, and consequently the results have a relatively low statistical power. Another limitation could appear the different ethnicity of the populations studies that could be considered as a further source of heterogeneity.

Considering these limitations, the results of the present systematic review should be considered with caution as compared to the available literature, in particular previously published systematic reviews of the literature. One narrative review of the literature published in 2010 by Fitzpatrick and Katz provided evidence of a significant association between periodontal disease and oral cancer while the evidence of a link to other types of cancer was questionable and controversial [13]. However, such conclusions were based on a narrative, although exhaustive, interpretation of the results without any attempt of meta-analysis, including studies relating cancer and tooth loss, thus incurring in the limitation exposed above.

With regard to narrative reviews, they reported a correlation between periodontitis (or periodontal diseases in general, since a heterogeneity among assessment of periodontal status was reported in the included papers) and head and neck cancer, even though the lack of a meta-analysis (due to the narrative nature of these papers) and the choice of inclusion criteria (broader that those used in the present review) could have limited the validity of the results [27, 56].

Other published meta-analyses on the same topic should be considered carefully when comparing to the results obtained in the present study. One paper reported the outcomes of one meta-analysis attempting to relate the prevalence rate of *P*. *gingivalis* and the development of cancer [21]. This study found that the prevalence of such bacteria increased the chance of cancer development by 1.36 times. It has to be considered that the reported CI (95%) for such OR was 0.47–3.07 based on a total of four studies, two supporting the association and two not supporting. Moreover, the test for overall effect was missing. So, the interpretation of the OR value should be made with extreme caution. The absence of a strong association between the presence of some bacteria and cancer was confirmed also by a paper published in 2016 reporting the result of a prospective study on postmenopausal women [57].

With regard to the sole oral cancer, the association with periodontitis was evaluated in a meta-analysis published by Yao and co-workers in 2014 [28]. A significant association was reported (OR = 3.53, 95% CI (1.52–8.23); *P =* 0.003) even though a substantial heterogeneity among the studies was found in all comparisons and this was probably due to the differences in the assessment methods, which were significantly different. This result confirmed the one obtained in another meta-analysis published by Zeng and colleagues in 2013 [58], albeit showing a consistent heterogeneity among the studies included in the quantitative synthesis. In fact, the hypothesis that local risk factors could be the cause of a relationship between periodontitis and oropharyngeal cancer than between other cancer types is still in need of a scientific support.

With regard to the relation between periodontitis and cancer mortality, one study that was not included in the present review, found some correlation with mortality for lung cancer, but the effect of potential confounders should be furtherly explored [59].

Considering the limitations, the present study found a low but statistically significant association between periodontitis and different types of cancer (both alone and pooled together). Despite the statistical significance, the clinical value (external validity) and the statistical power of this relationship were significantly limited by the low number of papers included in the quantitative synthesis, and we can speculate that the evidence of such correlation needs more support to be hypothesized and it is far to be considered conclusive.

In order to better understand the mechanisms and to explore the existence and the strength of the association between cancer and periodontitis more studies are needed, with standardized methods for periodontal evaluation, assessment and classification, and representative samples.

## Supporting information

S1 Table PRISMA checklist. (DOC)
